# Genome Size Estimation and Full-Length Transcriptome of *Sphingonotus tsinlingensis*: Genetic Background of a Drought-Adapted Grasshopper

**DOI:** 10.3389/fgene.2021.678625

**Published:** 2021-07-12

**Authors:** Lu Zhao, Hang Wang, Ping Li, Kuo Sun, De-Long Guan, Sheng-Quan Xu

**Affiliations:** College of Life Sciences, Shaanxi Normal University, Xi’an, China

**Keywords:** *Sphingonotus* Fieber, grasshoppers, PacBio isoform sequencing, gene functions, genetic background

## Abstract

*Sphingonotus* Fieber, 1852 (Orthoptera: Acrididae), is a grasshopper genus comprising approximately 170 species, all of which prefer dry environments such as deserts, steppes, and stony benchlands. In this study, we aimed to examine the adaptation of grasshopper species to arid environments. The genome size of *Sphingonotus tsinlingensis* was estimated using flow cytometry, and the first high-quality full-length transcriptome of this species was produced. The genome size of *S. tsinlingensis* is approximately 12.8 Gb. Based on 146.98 Gb of PacBio sequencing data, 221.47 Mb full-length transcripts were assembled. Among these, 88,693 non-redundant isoforms were identified with an N50 value of 2,726 bp, which was markedly longer than previous grasshopper transcriptome assemblies. In total, 48,502 protein-coding sequences were identified, and 37,569 were annotated using public gene function databases. Moreover, 36,488 simple tandem repeats, 12,765 long non-coding RNAs, and 414 transcription factors were identified. According to gene functions, 61 cytochrome P450 (CYP450) and 66 heat shock protein (HSP) genes, which may be associated with drought adaptation of *S. tsinlingensis*, were identified. We compared the transcriptomes of *S. tsinlingensis* and two other grasshopper species which were less tolerant to drought, namely *Mongolotettix japonicus* and *Gomphocerus licenti*. We observed the expression of CYP450 and HSP genes in *S. tsinlingensis* were higher. We produced the first full-length transcriptome of a *Sphingonotus* species that has an ultra-large genome. The assembly characteristics were better than those of all known grasshopper transcriptomes. This full-length transcriptome may thus be used to understand the genetic background and evolution of grasshoppers.

## Introduction

*Sphingonotus* Fieber, 1852 (Orthoptera: Acrididae), is a species-rich genus of grasshoppers that comprises 192 valid species and subspecies (Cigliano et al., 2017). This genus shows extensive radiation in the Palearctic, and many species are endemic to islands (Husemann et al., 2015). These grasshoppers are distributed in arid zones of the Northern Hemisphere, with diverse hotspots in the Mediterranean and Central and Eastern Asia (Dey et al., 2018). Single species have been recorded in the Galapagos Islands, Mexico, the Caribbean, Brazil, and northwestern Australia (Rentz, 1996; Cigliano et al., 2017). Their wide distribution, regional high incidence of endemism, and polymorphic stridulation organs make *Sphingonotus* an attractive study system for biogeographic and evolutionary questions (Johnsen, 1985; Benediktov, 2009; Husemann et al., 2011). The most intriguing characteristic of these species is that they prefer dry environments such as deserts, steppes, and dry stony benchlands (Husemann et al., 2014). Recently, several studies examined the phylogeography and evolution of this genus. However, it remains unclear how its species have adapted to drought environments and radiated to such richness. *Sphingonotus tsinlingensis* (Orthoptera: Acridoidea) is a grasshopper endemic to China, where it occurs on sandy and stony benchlands along the northern Qinling Mountains (Zheng et al., 1963). This species belongs to the oriental lineage of *Sphingonotus*, represents the adaptive status of *Sphingonotus spp.* in the East, and may serve as an ideal model for studying the evolution and ecology of grasshoppers in arid environments (Husemann et al., 2014; Moussi et al., 2018). Nonetheless, only few studies and sequences of respective functional genes of this species are available (Cui and Huang, 2012; Shah et al., 2019). Thus, further reference sequences and molecular genetic studies are required to elucidate its evolutionary and ecological characteristics.

The large genomes of Acrididae and widespread occurrence of repetitive elements entail challenges regarding genome assembly, and the sequencing costs are considerably high (Wang et al., 2014; Verlinden et al., 2020). Transcriptomics represent an alternative to genomic approaches for non-model organisms (Yuan et al., 2019). Published Acridid transcriptomes include those of *Gomphocerus sibiricus* (Shah et al., 2019), *Locusta migratoria* (Zhang et al., 2018), *Ceracris nigricornis* (Yuan et al., 2019), *Chorthippus biguttulus* (Berdan et al., 2017), *Shirakiacris shirakii* (Qiu et al., 2016), and *Xenocatantops brachycerus* (Zhao et al., 2018). Many available transcripts are associated with growth, development, environmental adaptability, and metabolism of nutrients and bioactive components, including candidate olfactory-related genes, pigmentation, and green pigment metabolism pathways.

Full-length transcriptome analysis can help identify coding and non-coding RNA and quantify differential gene expression. Moreover, it plays an important role in deciphering genomic functions with respect to physiological mechanisms and responses to environmental challenges (Jiang et al., 2015). Full-length transcriptomes of the two grasshoppers *Gomphocerus licenti* and *Mongolotettix japonicus* have been published previously, which yielded 590,112 and 566,165 circular consensus sequences (CCSs) as well as 458,131 and 428,979 full-length non-chimeric (FLNC) reads, respectively. In total, 17,970 and 16,766 unigenes were identified, with 17,495 and 16,373 coding sequences (CDSs), 1,082 and 813 transcription factors (TFs), 11,840 and 10,814 simple sequence repeats (SSRs), and 905 and 706 long non-coding RNAs (lncRNAs) by analyzing the transcriptomes of *G. licenti* and *M. japonicus*, respectively; 15,803 and 14,846 respective unigenes were annotated in public databases (Yuan et al., 2020).

In this study, the genome size (GS) and transcriptome of *S. tsinlingensis* were produced and examined for further functional and ecological studies using the deep-coverage PacBio isoform sequencing technique (Camacho et al., 2015; Yuan et al., 2019). In addition to protein-coding genes, different types of genetic elements such as TFs, SSRs, and lncRNAs were identified and classified. The produced full-length transcriptome of *S. tsinlingensis* was compared to published transcriptomes of other grasshoppers for quality evaluation, and heat shock protein (HSP) and cytochrome P450 (CYP450) genes were analyzed to investigate drought adaptation in this species.

## Materials and Methods

### Sample Collection

Six adult *S. tsinlingensis* males were collected from a natural population at a pebble beach in Xi’an (34°02′05.2″ N, 108°33′04.3″ E) on September 14, 2020. Some grasshoppers were kept alive in insect mesh cages until flow cytometry (FCM) was performed in the laboratory, while others were dissected, immediately immersed in liquid nitrogen, and stored at −80°C.

### GS Estimation Using FCM

Flow cytometry was used to investigate the GS of *S. tsinlingensis* (Dolezel and Bartos, 2005). DNA content is directly proportional to FCM fluorescence intensity. Thus, the GS of an organism can be calculated by comparison of a sample’s fluorescence intensity with that of an internal standard of known GS. *L. migratoria* (1C = 6.5 G) (Wang et al., 2014) was chosen as an internal standard. FCM was performed as previously described (Gregory and Johnston, 2008; Hare and Johnston, 2011). To prepare single cell suspensions, head tissues of *L. migratoria* and *S. tsinlingensis* were removed, placed in a tissue grinder with 1 mL Galbraith buffer (Galbraith et al., 1983), and ground 20 times. Then, the solution was filtered through a 38-μm nylon mesh to remove cellular debris and stained using 50 μg/mL propidium iodide. The above steps were performed on ice. The solution was then stored in the dark at 4°C for 30 min. The GS was assessed using a flow cytometer (Cytoflex S; Beckman Coulter, Krefeld, Germany) with three technical replicates, which were activated with a 488-nm laser and low flow rates.

### RNA Extraction

Total RNA was isolated from muscle tissues of the intact body of all individuals after removing the guts. Extractions were conducted using TRIzol reagent (Invitrogen, Carlsbad, CA, United States), following the manufacturer’s instructions. RNA degradation and contamination were screened using 1% agarose gel electrophoresis, and RNA integrity and purity were determined using an Agilent 2100 Bioanalyzer (Agilent Technologies, CA, United States) and a NanoDrop 2000 device (Thermo Scientific, Wilmington, DE, United States), respectively. Only total RNA samples with an RNA integrity index of ≥8 were used for producing high-throughput sequencing libraries.

### Library Preparation and Sequencing

The protocol of SMARTer PCR cDNA Synthesis Kit (TaKaRa, Dalian, China) was used to synthesize full-length cDNA and cDNA fractions. The BluePippin Size Selection system (Sage Science, Massachusetts, United States) was used to select the PCR products. SMRTbell (Pacific Biosciences, Menlo Park, CA, United States) template libraries were produced using the SMRTbell Template Prep Kit (Pacific Biosciences) according to the manufacturer’s instructions. An Agilent 2100 Bioanalyzer and a Qubit 2.0 device (Life Technologies, Carlsbad, CA, United States) were used to assess library quality and concentration, respectively. SMRT sequencing was performed using a PacBio Sequel platform (PacBio) at Novogene Technology Co. (Novogene, Beijing, China). An Illumina (San Diego, CA, United States) sequencing library was constructed using a Gene Expression Sample Prep Kit (Illumina), according to the manufacturer’s instructions. The qualified library was paired-end sequenced (2 × 150 bp) on an Illumina HiSeq X Ten (Illumina) platform by a commercial provider (Novogene).

### Data Analyses

PacBio data were processed using the SMRT Link 5.1 software pipeline (Pacific Biosciences). First, subreads were identified, and CCSs were produced using corrections between subreads. The CCSs were divided into FLNC and NFL sequences according to whether they contained 5’-primers, 3’-primers, and poly-A tails. FLNC reads were clustered using the ICE algorithm to obtain consensus sequences; Arrow software^1^ was used to refine consensus isoforms using the NFL to produce refined consensus sequences. Illumina RNA-seq short reads were filtered to remove adaptor sequences, ambiguous reads with “N” bases, and low-quality reads. Filtered Illumina data were then used to refine consensus sequences using Proovread (Hackl et al., 2014). Redundant isoforms (identity < 0.9; coverage age < 0.85) were eliminated using the CD-HIT program without considering the 5’-difference. To process raw Illumina sequencing data, Trimmomatic and FastQC (Poluri et al., 2019) software programs were used. HISAT (Pertea et al., 2016) was then used to map the filtered reads to the genome. Fragments per kilobase of exon per million fragments mapped (FPKM) values were calculated using StringTie (Pertea et al., 2015), and 25,761 genes had FPKM values.

### Gene Functional Annotation

Gene functions were annotated using the following databases: non-redundant protein sequences (NR) (Li et al., 2002), non-redundant nucleotide sequences (NT), Protein Family (Pfam), clusters of orthologous groups of proteins (KOG) (Tatusov et al., 2003), Swiss-Prot (Bairoch and Apweiler, 2000), Kyoto Encyclopedia of Genes and Genomes (KEGG) (Kanehisa et al., 2004), and Gene Ontology (GO) (Ashburner et al., 2000). The Basic Local Alignment Search Tool (BLAST) was used with an *e*-value of 1^–10^ in the NT database analysis, and the Diamond BLASTX software was used with an *e*-value 1^–10^ in the NR, KOG, Swiss-Prot, and KEGG database analyses. Hmmscan software was used for Pfam database analysis.

### Protein-CDS Prediction

ANGEL software (Shimizu et al., 2006) was used to determine CDSs among cDNA sequences. ANGEL calculates the coding potential of all codons using information from a short region around the target codon. Short regions are generated using a sliding window. All codons were labeled as CDS or ELSE, according to their coding potential. The most probable path was then traced using a Markov chain model with dynamic programming. Positions where a frame was changed were detected as the rough positions of frameshift errors in the path. Finally, each rough position was modified by selecting the most probable position from the candidate positions located near the rough positions.

### TF and lncRNA Analysis

Transcription factors were predicted using the AnimalTFDB 2.0 database^2^. Hmmscan software was used for comparison with the AnimalTFDB database to screen third-generation sequences (*e*-value < 0.0001) for TF identification and assign transcripts to different families. The Coding–Non-Coding Index (Sun et al., 2013), coding potential calculator (Kong et al., 2007), Pfam-scan (Finn et al., 2016), and predictors of long non-coding RNAs and messenger RNAs based on an improved k-mer scheme (Li et al., 2014) tools were used to predict the coding potential of transcripts. Transcripts with predicted coding potential according to all four tools were filtered out, and those without coding potential constituted the candidate set of lncRNAs.

### SSR Analysis

Simple sequence repeats of the transcriptome were identified using MIcroSAtellite^3^, which allows the identification and localization of perfect microsatellites as well as compound microsatellites that are interrupted by a certain number of bases.

## Results

### GS Estimation of *S. tsinlingensis*

The GS of *S. tsinlingensis* was estimated using FCM. Two peaks were identified, with P1 and P2 representing the counted cell intensities of *L. migratoria* and *S. tsinlingensis*, respectively. The mean fluorescent intensities of peaks P1 and P2 were 8.11 × 10^5^ and 15.98 × 10^5^ (low coefficient of variation, less than 5%), respectively. The ratio of GS of *S. tsinlingensis* to *L. migratoria* was equal to that of P2 to P1. The GS of *S. tsinlingensis* was calculated to be approximately 12.81 Gb (Supplementary Figure 1). This GS was considerably larger than that of *L. migratoria* and was one of the largest among Acrididae.

### Full-Length Transcriptome Assembly of *S. tsinlingensis*

In total, 146.98 Gb raw polymerase reads were obtained using the PacBio isoform sequencing platform. After filtering and self-correcting the raw data, 28.6 million subreads were processed into 901,383 CCS reads. These CCSs were then clustered and polished into 88,693 non-redundant full-length, non-chimeric isoforms, which was the final molecular sequence pool for screening gene components (Supplementary Table 1; Supplementary Figure 2). The full-length transcriptome of *S. tsinlingensis* showed better quality characteristics than other reported grasshopper genomes and transcriptomes. The transcript number was 88,693, and the percentage of long non-redundant isoforms (over 1,000 bp) was 99.28%. The most outstanding parameter was an average length of 2,497 bp and N50 value of 2,726 bp, which was longer than other transcriptomes (Table 1).

**TABLE 1:** Comparison of transcriptome assemblies and gene numbers with the eight published Acrididae transcriptomes.

Species name	Transcript length (bp)	Transcript number	Average length (bp)	N50 (bp)	Genome size (Gb)
***Gomphocerus sibiricus***	86,939,307 Shah et al., 2019	82,251	1,057	1,357	8.95 Gosalvez et al., 1980
***Gomphocerus licenti***	136,517,140 Yuan et al., 2020	96,643	1412	2,371	∼9 Gosalvez et al., 1980
***Mongolotettix japonicus***	199,205,336 Yuan et al., 2020	126,643	1572	2,671	N.A.
***Shirakiacris shirakii***	39,306,387 Qiu et al., 2016	135,320	290	428	8.55∼8.96 John and Hewitt, 1966; Fox, 1970
***Locusta migratoria***	392,472,062 Zhang et al., 2018	607,901	646	N.A.	5.28∼6.44 Bier and Muller, 1969; Wang et al., 2014
***Ceracris nigricornis***	112,816,350 Yuan et al., 2019	70,581	1598.4	2,434	N.A.
***Xenocatantops brachycerus***	16,884,056 Zhao et al., 2018	27,004	625	1,031	N.A.
***Chorthippus biguttulus***	212,567,026 Berdan et al., 2017	1,564,070	478	424	∼10 John and Hewitt, 1966; Wilmore and Brown, 1975
***Sphingonotus tsinlingensis***	221,466,421	88,693	2,497	2,726	12.81

*NA, means not available.*

### Prediction of CDSs

**FIGURE 1:**
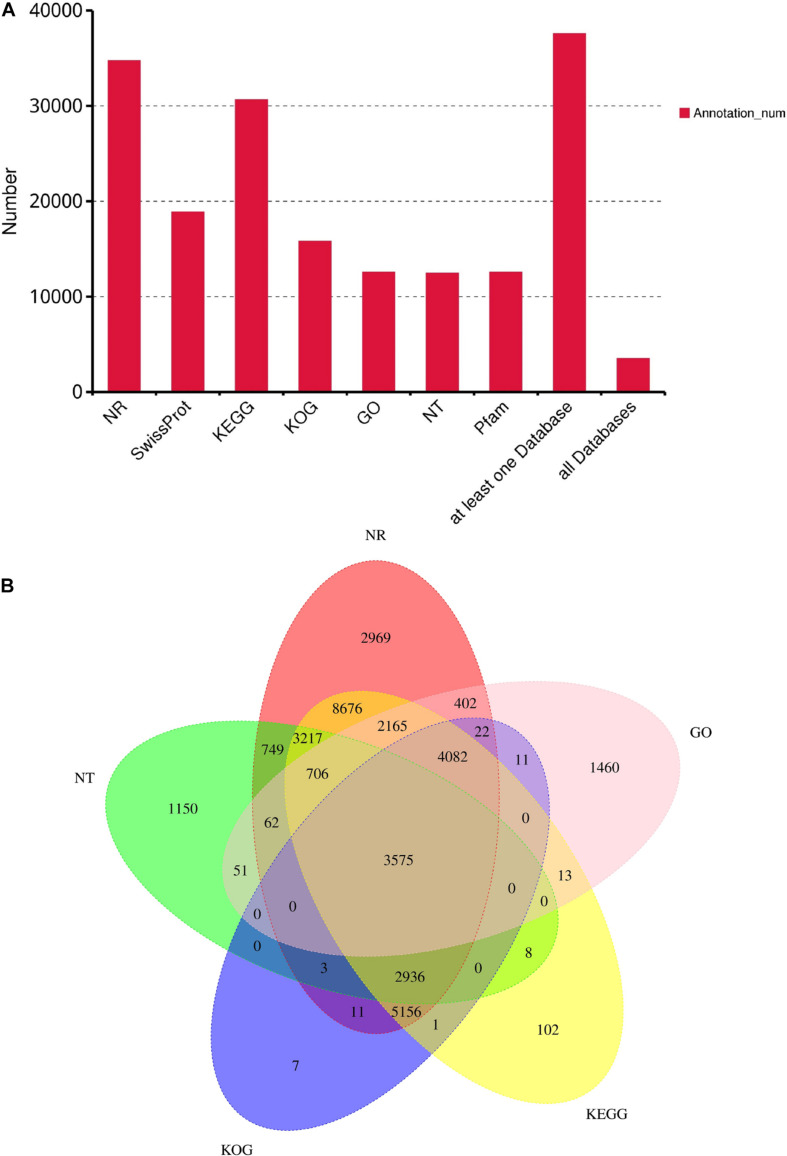
Statistics on the functional annotation of all *Sphingonotus tsinlingensis* protein-coding sequences (CDSs) in seven common databases **(A)**, and a Venn diagram showing the functional annotation of *S. tsinlingensis* transcripts in the five most commonly used databases **(B)**. NR, non-redundant protein sequence; KEGG, Kyoto Encyclopedia of Genes and Genomes; KOG, EuKaryotic Orthologous Groups; GO, gene ontology; NT, non-redundant nucleotide sequences; Pfam, protein family.

Among full-length, non-chimeric isoforms of *S. tsinlingensis*, 48,502 transcripts with CDSs were identified, accounting for approximately 54.68% of the total isoforms. The N50 length of CDSs was 1,230 bp, and each CDS encoded 229.3 amino acids on average (Supplementary Figure 3). In total, 37,569 CDSs were retrieved after integrated annotations using seven gene databases, accounting for 78.18% of the total CDSs (Figure 1A; Supplementary Table 2). A Venn diagram was generated to visualize the respective contribution of different databases to the annotations, and 3,575 genes were annotated by the five most commonly employed databases (Figure 1B).

The main annotations were from the NR database (34,731 genes). Among these, *S. tsinlingensis* produced the most hits with *Zootermopsis nevadensis* (12.86%, 4,459 genes; Supplementary Figure 4A). The pattern of hits suggested that these species shared close phylogenetic positions, and the percentages corresponded to the number of their indexed sequences in the database, which also revealed that the proportion of Acrididae was not as high as that of other species. GO and KEGG annotations were used to describe the genome composition of *S. tsinlingensis*. Cells and cell parts (2,865 genes) were the most represented terms in the cell component categories, and binding (7,457 genes) was the most represented molecular function (Supplementary Figure 4B). In the KEGG annotations, 30,637 annotations were assigned to 355 signaling pathways. The most enriched KEGG pathways were associated with basic metabolism processes, including signal transduction (1,008 genes), transport and catabolism (608 genes), amino acid metabolism (606 genes), cancer overview (578 genes), and endocrine system (571 genes; Supplementary Figure 4C). Several highly conserved signaling pathways that play a critical role in insect growth and body development were markedly enriched, including the Wnt, Notch, transforming growth factor-β, Janus kinase/signal transducer and activator of transcription, mitogen-activated protein kinase, and Hedgehog pathways.

### CYP450 and HSP Genes

Drought adaption-related genes and their annotations were identified from the isoform sequences of *S. tsinlingensis*, including 61 xenobiotic CYP450 and 66 HSP genes. The lengths of CYP450 and HSP sequences varied from 1,019 to 5,346 bp and from 1,438 to 4,428 bp, respectively. Most BLAST identities of these target-focused sequences were below 95%, which suggested that they were novel to the current understanding of genetic mechanisms in *S. tsinlingensis* (Supplementary Figure 5).

**FIGURE 2:**
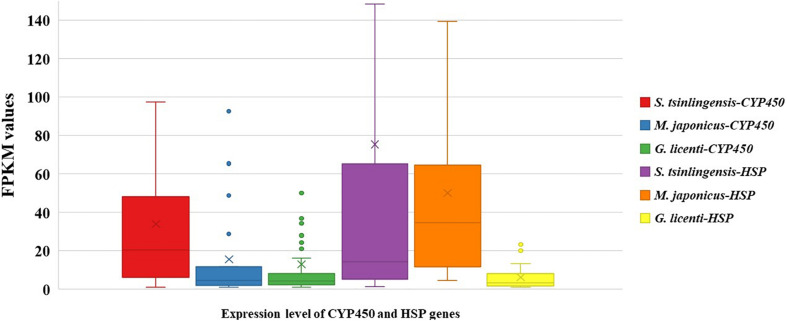
Expression levels of CYP450 and HSP genes in *Sphingonotus tsinlingensis*, *Mongolotettix japonicus*, and *Gomphocerus licenti.* The *y*-axis indicates FPKM values.

To confirm that these sequences were associated with adaptation to drought in *S. tsinlingensis*, we compared the expression of CYP450 and HSP genes among the transcriptomes of *S. tsinlingensis* and two other grasshopper species that were less tolerant to dry areas (*M. japonicus* and *G. licenti*). The box plot illustrates that the distribution of expressions had a wider range with respect to CYP450 and HSP genes in *S. tsinlingensis* than in *M. japonicus* and *G. licenti*. In particular, the distribution ranges Q1 to Q3 of the FPKM values of CYP450 and HSP genes were 6.38 to 48.10 and 34 to 65.36, respectively, in *S. tsinlingensis*. However, these values were only 2.12 to 11.74 (CYP450) and 11.89 to 64.53 (HSP) in *M. japonicus* and 2.32 to 8.30 (CYP450) and 1.67 to 8.05 (HSP) in *G. licenti*, respectively (Figure 2). These results suggested that more members of CYP450 and HSP genes in *S. tsinlingensis* participated and contributed to its adaptation to drought. In addition, the overall expression levels of CYP450 and HSP genes were higher than those in other grasshoppers such as *M. japonicus* and *G. licenti.*

### LncRNA Identification

**FIGURE 3:**
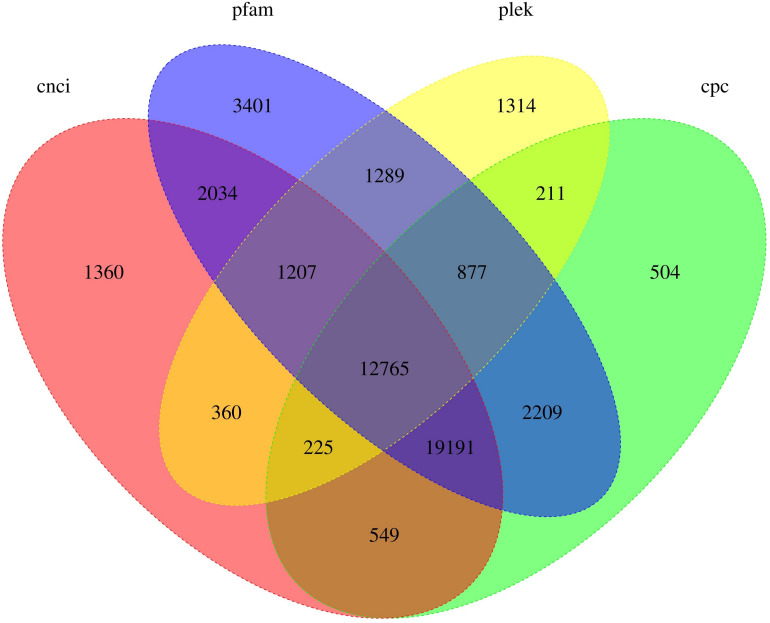
Venn diagram showing the distribution of long non-coding RNAs (lncRNAs) identified in *Sphingonotus tsinlingensis*. CNCI, Coding–Non-Coding Index; Pfam, protein family; PLEK, predictor of long non-coding RNAs and messenger RNAs based on an improved *k*-mer scheme; CPC, coding potential calculator.

LncRNAs were identified using a combination of the Coding Potential Calculator, Coding–Non-Coding Index, Coding Potential Assessment Tool, and Pfam methods. In total, 12,765 (22.94%) valid lncRNAs (>200 bp long and with more than two exons) were identified (Figure 3). A length distribution analysis of lncRNAs revealed that their lengths ranged from 212 to 8,365 bp, with a mean length of 2,090 bp. The N50 length of these identified lncRNAs was 2,185 bp.

The number of lncRNAs was significantly lower than that of mRNAs. To verify the two possible conditions, i.e., each type of lncRNA regulated multiple mRNAs or the valid lncRNA was not fully determined, the linkages between the 12,765 lncRNAs and 48,502 CDSs were checked. The lncRNAs were only related to 9,347 CDSs, suggesting that lncRNAs were not fully sequenced. Four HSP and no CYP450 gene-associated lncRNAs were identified, suggesting that the regulatory mechanisms of these HSP and CYP450 genes were not well characterized.

### TF Identification

**FIGURE 4:**
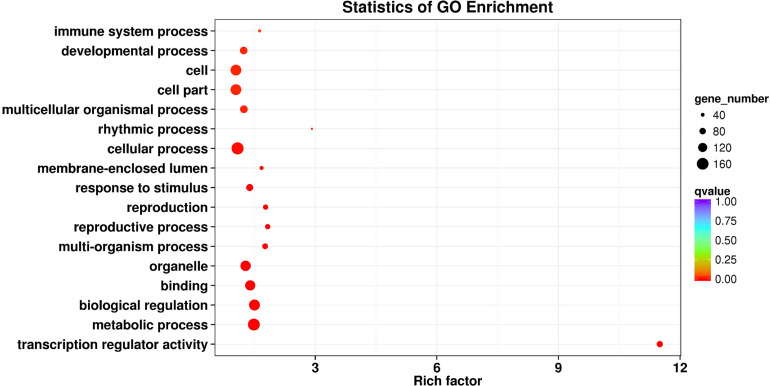
Gene ontology (GO) enrichment reveals the functions of genes that have been determined to be transcription factors (TFs). The rich factor refers to the ratio of the number of differentially expressed transcripts to the total number of annotated transcripts located in the GO term, and the *Q*-value is the *P*-value corrected by multiple hypothesis tests, with a value range of 0 to 1. The closer the *Q*-value is to zero, the more significant is the enrichment.

Transcription factors participate in gene expression regulation by linking lncRNAs and mRNAs. In this study, 414 putative TFs belonging to 38 TF gene families were predicted (Supplementary Table 3). zf-C2H2 (20.29%, 84/414) was the most abundant TF family, followed by ZBTB (16.18%, 67/414) and THAP (15.22%, 63/414; Supplementary Figure 6). The relationships among lncRNAs, TFs, and mRNAs are not discussed here because of the limited number of TFs. Instead, a GO enrichment analysis was conducted using genes in which TFs have been determined. Although lower in numbers, these genes did not present functional bias and covered almost all basic metabolic functions. Survival-dependent mechanisms such as multicellular organismal processes, developmental processes, and immune system processes were the most enriched mechanisms (Figure 4).

### SSR Motif Analysis

Simple sequence repeats are important molecular markers that indicate genetic polymorphisms. In total, 36,488 SSRs were identified among all isoforms of *S. tsinlingensis*; 3,719 genes contained more than one SSR, and 328 SSRs were present in the compound form. Among the six SSR types, mono-nucleotide repeats (18,078, 49.55%) were most abundant, followed by dinucleotide repeats (9,859, 27.02%), trinucleotide repeats (7,612, 20.86%), tetranucleotide repeats (791, 2.17%), pentanucleotide repeats (107, 0.29%), and hexanucleotide repeats (41, 0.11%; Supplementary Figure 7). This full-length transcriptome of *S. tsinlingensis* contained more SSRs than the other published full-length transcriptomes of grasshoppers, including that of *G. licenti*, *M. japonicus*, and *S. shirakii*, possibly because of higher heterozygosity of *S. tsinlingensis* or a more thorough SSR search.

## Discussion

To the best of our knowledge, this is the first genomic study on *Sphingonotus* spp. Although *S. tsinlingensis* is an oriental species endemic to China, it represents the most popular morphological type of the grasshopper genus *Sphingonotus* and the subgenus *Spingonotus*. These taxa show hind wings with a curved black bind and blue color at the base (Zheng et al., 1963). A comprehensive transcriptome comprises all expressed gene sequences of an organism. These sequences can act as gene pools to provide and complement species-specific sequences. In addition, full-length transcriptome has better connectivity and integrity compared to Illumina short reads, and play an important role in gene annotation. Therefore, the transcriptome of *S. tsinlingensis* may provide a reference for the study of *Sphingonotus* species. Data, including lncRNAs, TFs, and SSRs, may be used as a genetic background for gene identification, homologous gene screening, phylogenetics, and adaptive evolution analysis. Moreover, through well-connected and accurate sequences and short-read quantitative analyses, these elements were found to be involved in the unique physiological processes of this species. These results suggest that our data are reliable and may be of use for future studies.

Based on the ultra-large GS, we suggest using full-length transcriptomes instead of whole genomes when conducting genomic studies on grasshoppers of this genus. In general, PacBio sequences are 85% accurate at 30-fold coverage of the transcriptome (Midha et al., 2019). In this study, the full-length transcriptome was successfully produced at 60-fold coverage. The success of this strategy will help optimize the experimental design for similar future studies. Because grasshoppers have very large genomes and genome assemblies are difficult to construct owing to the highly repetitive regions (Schatz et al., 2010; Shah et al., 2019), grasshopper genome assembly requires a considerable amount of sequencing and computation and is thus time-consuming and expensive. Even with respect to mRNA, sequencing frequently fails to enrich effective sequences owing to the large GS, and rare genes are missed because of sequencing bias (Gao et al., 2016). To address this problem, sequencing depth was increased in the current study to produce a sufficient amount of effective sequences. The number of protein-expressing genes in the functional gene dataset observed in the current study was the same as that found in previous studies (Yuan et al., 2020). The proportion of annotated functional genes was increased, and the average length of genes was markedly improved. From the composition of gene functions, gene detection was complete, and many basic metabolism-related genes were detected. Among grasshopper species for which both transcriptome and genome was reported, the GS of *S. tsinlingensis* was the largest. Its GS of approximately 12.81 Gb was almost twice that of *L. migratoria* (Wang et al., 2014). A comprehensive exploration of the effective sequences in the genome was conducted when the ratio of transcript size to GS was less than 2%.

These results suggest that the full-length transcriptome is an effective method for studying the genomics of grasshopper species with ultra-large and complex genomes. However, there are some limitations to this study. For example, lncRNAs, miRNAs, TFs, and mRNAs obtained from the full-length transcriptome typically play important roles in regulating gene expression at epigenetic, transcriptional, and post-transcriptional levels and constructing miRNA-lncRNAs-mRNAs-TF networks (Ye et al., 2018). Nonetheless, no association between these four regulatory elements was identified in the present study. The regulatory elements detected using functional enrichment reflected only the basic metabolic processes. To obtain better data in future studies and better enrich the results of the full-length transcriptome, a combination of multi-omics methods should be used, such as competing endogenous RNA for identification (Jiang et al., 2020) supplemented with mRNA next-generation sequencing for expression profile analysis.

This is the first GS and full-length transcriptome study on a species of the genus *Sphingonotus*. *S. tsinlingensis* has an ultra-large genome (12.81 Gb). Deep PacBio sequencing is currently the best method for retrieving nucleic acid sequences from species with ultra-large and complex genomes. The full-length transcriptome produced in the present study provides a reference resource for future studies on gene identification and comparison and will help improve our understanding of the mechanisms by which grasshoppers adapt to arid environments.

## Data Availability Statement

The datasets presented in this study can be found in online repositories. The names of the repository/repositories and accession number(s) can be found below: https://www.ncbi.nlm.nih.gov/, PRJNA707366 and https://zenodo.org/record/4588269#.YEdESKSwM3s, 4588269.

## Author Contributions

LZ, D-LG, and S-QX conceived the study and designed the experiments. D-LG analyzed the data. LZ wrote the manuscript. LZ, HW, PL, and KS performed the sequencing experiments. D-LG and S-QX revised the manuscript. All authors read and approved the final manuscript.

## Conflict of Interest

The authors declare that the research was conducted in the absence of any commercial or financial relationships that could be construed as a potential conflict of interest.

## Footnotes

1 https://www.pacb.com/wp-content/uploads/SMRT_Tools_Reference_Guide_v600.pdf

2 http://bioinfo.life.hust.edu.cn/AnimalTFDB/

3 https://webblast.ipk-gatersleben.de/misa/

## References

[B1] Ashburner M. Ball C. A. Blake J. A. Botstein D. Butler H. Cherry J. M. (2000). Gene ontology: tool for the unification of biology. The Gene Ontology Consortium. *Nat. Genet.* 25 25–29. 10.1038/75556 10802651 PMC3037419

[B2] Bairoch A. Apweiler R. (2000). The SWISS-PROT protein sequence database and its supplement TrEMBL in 2000. *Nucleic Acids Res.* 28 45–48. 10.1093/nar/28.1.45 10592178 PMC102476

[B3] Benediktov A. A. (2009). To the taxonomy and bioacoustics of grasshoppers of the genus *Sphingonotus* Fieber, 1852 (Orthoptera, *Acrididae*, *Oedipodinae*). *Proc. Russ. Entomol. Soc.* 80 21–33. 10.3897/jor.26.14550

[B4] Berdan E. L. Finck J. Johnston P. R. Waurick I. Mazzoni C. J. Mayer F. (2017). Transcriptome profiling of ontogeny in the acridid grasshopper *Chorthippus biguttulus*. *PLoS One* 12:e0177367. 10.1371/journal.pone.0177367 28520760 PMC5435247

[B5] Bier K. Muller W. J. B. Z. (1969). DNS-Messungen bei Insekten und eine Hypothese uber retardierte Evolution and besonderen DNS-Reichtum im Tierreich. *Biol. Zent. Bl.* 88 425–449. 10.1007/978-3-642-49227-3_29

[B6] Camacho J. P. Ruiz-Ruano F. J. Martín-Blázquez R. López-León M. D. Cabrero J. Lorite P. (2015). A step to the gigantic genome of the desert locust: chromosome sizes and repeated DNAs. *Chromosoma* 124 263–275. 10.1007/s00412-014-0499-0 25472934

[B7] Cigliano M. M. Braun H. Eades D. C. Otte D. (2017). *Orthoptera Species File. Version 5.0/5.0.* Available Online at: http://Orthoptera.SpeciesFile.org [accessed Dec 11, 2017].

[B8] Cui A. M. Huang Y. (2012). [Phylogenetic relationships among Orthoptera insect groups based on complete sequences of 16S ribosomal RNA]. *Yi Chuan* 34 597–608. 10.3724/sp.j.1005.2012.00597 22659432

[B9] Dey L. S. Saboori A. Hodjat S. H. Tork M. Pahlow F. Husemann M. (2018). A faunistic review of the Iranian species of *Sphingonotus* (Orthoptera, *Oedipodinae*) with an online key to species. *Zootaxa* 4379 151–176. 10.11646/zootaxa.4379.2.1 29689982

[B10] Dolezel J. Bartos J. (2005). Plant DNA flow cytometry and estimation of nuclear genome size. *Ann. Bot.* 95 99–110. 10.1093/aob/mci005 15596459 PMC4246710

[B11] Finn R. D. Coggill P. Eberhardt R. Y. Eddy S. R. Mistry J. Mitchell A. L. (2016). The Pfam protein families database: towards a more sustainable future. *Nucleic Acids Res.* 44 D279–85. 10.1093/nar/gkv1344 26673716 PMC4702930

[B12] Fox D. P. J. C. (1970). A non-doubling DNA series in somatic tissues of the locusts *Schistocerca gregaria* (Forskål) and *Locusta migratoria* (Linn.). *Chromosoma* 29 446–461. 5480729 10.1007/BF00281927

[B13] Galbraith D. W. Harkins K. R. Maddox J. M. Ayres N. M. Sharma D. P. Firoozabady E. (1983). Rapid flow cytometric analysis of the cell cycle in intact plant tissues. *Science* 220 1049–1051. 10.1126/science.220.4601.1049 17754551

[B14] Gao R. Yu K. Nie J. Lian T. Jin J. Liljas A. (2016). Deep sequencing reveals global patterns of mRNA recruitment during translation initiation. *Sci. Rep.* 6:30170. 10.1038/srep30170 27460773 PMC4962057

[B15] Gosalvez J. López-Fernandez C. Esponda P. J. C. (1980). Variability of the DNA Content in Five Orthopteran Species. *Caryologia* 33 275–281. 10.1080/00087114.1980.10796840

[B16] Gregory T. R. Johnston J. S. (2008). Genome size diversity in the family Drosophilidae. *Heredity (Edinb)* 101 228–238. 10.1038/hdy.2008.49 18523443

[B17] Hackl T. Hedrich R. Schultz J. Förster F. (2014). proovread: large-scale high-accuracy PacBio correction through iterative short read consensus. *Bioinformatics* 30 3004–3011. 10.1093/bioinformatics/btu392 25015988 PMC4609002

[B18] Hare E. E. Johnston J. S. (2011). Genome size determination using flow cytometry of propidium iodide-stained nuclei. *Methods Mol. Biol.* 772 3–12. 10.1007/978-1-61779-228-1_1 22065429

[B19] Husemann M. Deppermann J. Hochkirch A. (2014). Multiple independent colonization of the Canary Islands by the winged grasshopper genus *Sphingonotus* Fieber, 1852. *Mol. Phylogenet. Evol.* 81 174–181. 10.1016/j.ympev.2014.09.017 25256055

[B20] Husemann M. Habel J. C. Namkung S. Hochkirch A. Otte D. Danley P. D. (2015). Molecular evidence for an old world origin of Galapagos and Caribbean band-winged grasshoppers (*Acrididae: Oedipodinae*: *Sphingonotus*). *PLoS One* 10:e0118208. 10.1371/journal.pone.0118208 25692768 PMC4334964

[B21] Husemann M. Ray J. Hochkirch A. J. Z. (2011). A revision of the subgenus Parasphingonotus Benediktov & Husemann, 2009 (Orthoptera: *Oedipodinae*: Sphingonotini). *Zootaxa* 2916 51–61. 10.11646/zootaxa.2916.1.4

[B22] Jiang J. Bi Y. Liu X. P. Yu D. Yan X. Yao J. (2020). To construct a ceRNA regulatory network as prognostic biomarkers for bladder cancer. *J. Cell Mol. Med.* 24 5375–5386. 10.1111/jcmm.15193 32233022 PMC7205833

[B23] Jiang Z. Zhou X. Li R. Michal J. J. Zhang S. Dodson M. V. (2015). Whole transcriptome analysis with sequencing: methods, challenges and potential solutions. *Cell. Mol. Life Sci.* 72 3425–3439. 10.1007/s00018-015-1934-y 26018601 PMC6233721

[B24] John B. Hewitt G. M. (1966). Karyotype stability and DNA variability in the *Acrididae*. *Chromosoma* 20 155–172. 10.1007/bf00335205

[B25] Johnsen P. (1985). Contributions to the knowledge of the genera *Sphingonotus*, Pseudosphingonotus and Wernerella in Africa, with description of four new species (*Acrididae: Oedipodinae*). *Natura Jutlandica* 21 149–168.

[B26] Kanehisa M. Goto S. Kawashima S. Okuno Y. Hattori M. (2004). The KEGG resource for deciphering the genome. *Nucleic Acids Res.* 32 D277–D280. 10.1093/nar/gkh063 14681412 PMC308797

[B27] Kong L. Zhang Y. Ye Z. Q. Liu X. Q. Zhao S. Q. Wei L. (2007). CPC: assess the protein-coding potential of transcripts using sequence features and support vector machine. *Nucleic Acids Res.* 35 W345–W349. 10.1093/nar/gkm391 17631615 PMC1933232

[B28] Li A. Zhang J. Zhou Z. (2014). PLEK: a tool for predicting long non-coding RNAs and messenger RNAs based on an improved k-mer scheme. *BMC Bioinformatics* 15:311. 10.1186/1471-2105-15-311 25239089 PMC4177586

[B29] Li W. Jaroszewski L. Godzik A. (2002). Tolerating some redundancy significantly speeds up clustering of large protein databases. *Bioinformatics* 18 77–82. 10.1093/bioinformatics/18.1.77 11836214

[B30] Midha M. K. Wu M. Chiu K. P. (2019). Long-read sequencing in deciphering human genetics to a greater depth. *Hum. Genet.* 138 1201–1215. 10.1007/s00439-019-02064-y 31538236

[B31] Moussi A. Dey L. S. Petit D. Abba A. Klesser R. Husemann M. J. A. Z. (2018). First genetic data for band-winged grasshoppers (Orthoptera: *Acrididae: Oedipodinae*) of the Biskra region of Algeria with new records for the country. *Afr. Zool.* 53 31–40. 10.1080/15627020.2018.1463172

[B32] Pertea M. Kim D. Pertea G. M. Leek J. T. Salzberg S. L. (2016). Transcript-level expression analysis of RNA-seq experiments with HISAT, StringTie and Ballgown. *Nat. Protoc.* 11 1650–1667. 10.1038/nprot.2016.095 27560171 PMC5032908

[B33] Pertea M. Pertea G. M. Antonescu C. M. Chang T. C. Mendell J. T. Salzberg S. L. (2015). StringTie enables improved reconstruction of a transcriptome from RNA-seq reads. *Nat. Biotechnol.* 33 290–295. 10.1038/nbt.3122 25690850 PMC4643835

[B34] Poluri R. T. K. Beauparlant C. J. Droit A. Audet-Walsh É (2019). RNA sequencing data of human prostate cancer cells treated with androgens. *Data Brief* 25:104372. 10.1016/j.dib.2019.104372 31485472 PMC6715830

[B35] Qiu Z. Liu F. Lu H. Yuan H. Zhang Q. Huang Y. (2016). *De Novo* Assembly and Characterization of the Transcriptome of Grasshopper Shirakiacris shirakii. *Int. J. Mol. Sci.* 17:1110. 10.3390/ijms17071110 27455245 PMC4964485

[B36] Rentz D. C. (1996). Grasshopper country: the abundant orthopteroid insects of Australia. *Q. Rev. Biol.* 72:337. 10.1086/419901

[B37] Schatz M. C. Delcher A. L. Salzberg S. L. (2010). Assembly of large genomes using second-generation sequencing. *Genome Res.* 20 1165–1173. 10.1101/gr.101360.109 20508146 PMC2928494

[B38] Shah A. Hoffman J. I. Schielzeth H. (2019). Transcriptome assembly for a colour-polymorphic grasshopper (Gomphocerus sibiricus) with a very large genome size. *BMC Genomics* 20:370. 10.1186/s12864-019-5756-4 31088494 PMC6518663

[B39] Shimizu K. Adachi J. Muraoka Y. (2006). ANGLE: a sequencing errors resistant program for predicting protein coding regions in unfinished cDNA. *J. Bioinform. Comput. Biol.* 4 649–664. 10.1142/s0219720006002260 16960968

[B40] Sun L. Luo H. Bu D. Zhao G. Yu K. Zhang C. (2013). Utilizing sequence intrinsic composition to classify protein-coding and long non-coding transcripts. *Nucleic Acids Res.* 41:e166. 10.1093/nar/gkt646 23892401 PMC3783192

[B41] Tatusov R. L. Fedorova N. D. Jackson J. D. Jacobs A. R. Kiryutin B. Koonin E. V. (2003). The COG database: an updated version includes eukaryotes. *BMC Bioinformatics* 4:41. 10.1186/1471-2105-4-41 12969510 PMC222959

[B42] Verlinden H. Sterck L. Li J. Li Z. Yssel A. Gansemans Y. (2020). First draft genome assembly of the desert locust, *Schistocerca gregaria*. *F1000Res* 9:775. 10.12688/f1000research.25148.1 PMC7607483 33163158

[B43] Wang X. Fang X. Yang P. Jiang X. Jiang F. Zhao D. (2014). The locust genome provides insight into swarm formation and long-distance flight. *Nat. Commun.* 5:2957. 10.1038/ncomms3957 24423660 PMC3896762

[B44] Wilmore P. J. Brown A. K. J. C. (1975). Molecular properties of orthopteran DNA. *Chromosoma* 51 337–345. 10.1007/bf00326320 1175452

[B45] Ye Y. Li S. L. Wang S. Y. (2018). Construction and analysis of mRNA, miRNA, lncRNA, and TF regulatory networks reveal the key genes associated with prostate cancer. *PLoS One* 13:e0198055. 10.1371/journal.pone.0198055 30138363 PMC6107126

[B46] Yuan H. Chang H. Zhao L. Yang C. Huang Y. (2019). Sex- and tissue-specific transcriptome analyses and expression profiling of olfactory-related genes in Ceracris nigricornis Walker (Orthoptera: *Acrididae*). *BMC Genomics* 20:808. 10.1186/s12864-019-6208-x 31694535 PMC6836668

[B47] Yuan H. Zhang X. Zhao L. Chang H. Yang C. Qiu Z. (2020). Characterization and analysis of full-length transcriptomes from two grasshoppers, Gomphocerus licenti and Mongolotettix japonicus. *Sci. Rep.* 10:14228. 10.1038/s41598-020-71178-5 32848169 PMC7450073

[B48] Zhang X. Kang X. Wu H. Silver K. Zhang J. Ma E. (2018). Transcriptome-wide survey, gene expression profiling and exogenous chemical-induced transcriptional responses of cytochrome P450 superfamily genes in migratory locust (*Locusta migratoria*). *Insect Biochem. Mol. Biol.* 100 66–77. 10.1016/j.ibmb.2018.06.006 29959977

[B49] Zheng Z. M. Tu Q. Liang L. Q. (1963). *A New Species of the Genus Sphingonotus Fieb. from China (Orthoptera: Acrididae)*. Acta Zoologica Sinica, 15 279–281.

[B50] Zhao L. Zhang X. Qiu Z. Huang Y. (2018). *De Novo* Assembly and Characterization of the Xenocatantops brachycerus Transcriptome. *Int. J. Mol. Sci.* 19:520. 10.3390/ijms19020520 29419810 PMC5855742

