# Supplementary material for: Genome Size Estimation and Full-Length Transcriptome of Sphingonotus tsinlingensis: Genetic Background of a Drought-Adapted Grasshopper
Source: Front Genet. 2021 Jul 12;12:678625. doi: 10.3389/fgene.2021.678625 (PMC8313316; doi:10.3389/fgene.2021.678625)
Supplement: Supplementary file 1 [file Data_Sheet_1.docx]

Supplementary Material

# Supplementary Tables

**Table S1.** Summary for the full-length transcriptome of *S. tsinlingensis*.

| Classification | Number | Average length (bp) | N50 (bp) |
| --- | --- | --- | --- |
| Polymerase Reads | 994,900 | 66,247 | 119,072 |
| Subreads | 28,612,043 | 2,229 | 2,410 |
| CCS | 901,383 | 2,556 | 2,770 |
| FLNC | 712,109 | 2,433 | 2,662 |
| Non-redundant isoforms | 88,693 | 2,497 | 2,726 |

CCS: circular consensus sequences; FLNC read: full-length non-chimeric read.

**Table S2.** Statistics of functional annotations of full-length transcriptome in public databases.

| Database | NR | SwissProt | KEGG | KOG | GO | NT | Pfam |
| --- | --- | --- | --- | --- | --- | --- | --- |
| Annotation  number | 34,731 | 18,866 | 30,637 | 15,804 | 12,549 | 12,457 | 12,549 |

**Table S3.** Statistics of transcription factors from 38 TF families.

| Transcription factors | Number |
| --- | --- |
| \| zf-C2H2 \| \| --- \| \| ZBTB \| \| THAP \| \| HTH \| \| TF_bZIP \| \| bHLH \| \| HMG \| \| zf-BED \| \| CSD \| \| MYB \| \| MBD \| \| C/EBP \| \| MH1 \| \| CP2 \| \| RHD \| \| Fork \| \| ARID \| \| PAX \| \| THR-like \| \| zf-GATA \| \| STAT \| \| TSC22 \| \| Homeobox \| \| ESR-like \| \| P53 \| \| HSF \| \| AF-4 \| \| zf-LITAF-like \| \| zf-MIZ  ETS  COE \| \| CBF  GCNF-like  NF-YB  NF-YC  RXR-like  SRF  RFX \| | \| 84 \| \| --- \| \| \| 67 \| \| --- \| \| 63 \| \| 28 \| \| 22 \| \| 18 \| \| 13 \| \| 13 \| \| 11 \| \| 8 \| \| 7 \| \| 6 \| \| 6 \| \| 6 \| \| 5 \| \| 5 \| \| 4 \| \| 4 \| \| 4 \| \| 4 \| \| 3 \| \| 3 \| \| 3 \| \| 3 \| \| 3 \| \| 3 \| \| 2 \| \| 2 \| \| 2  2  2  1  1  1  1  1  1  1 \| \| |

# Supplementary Figures


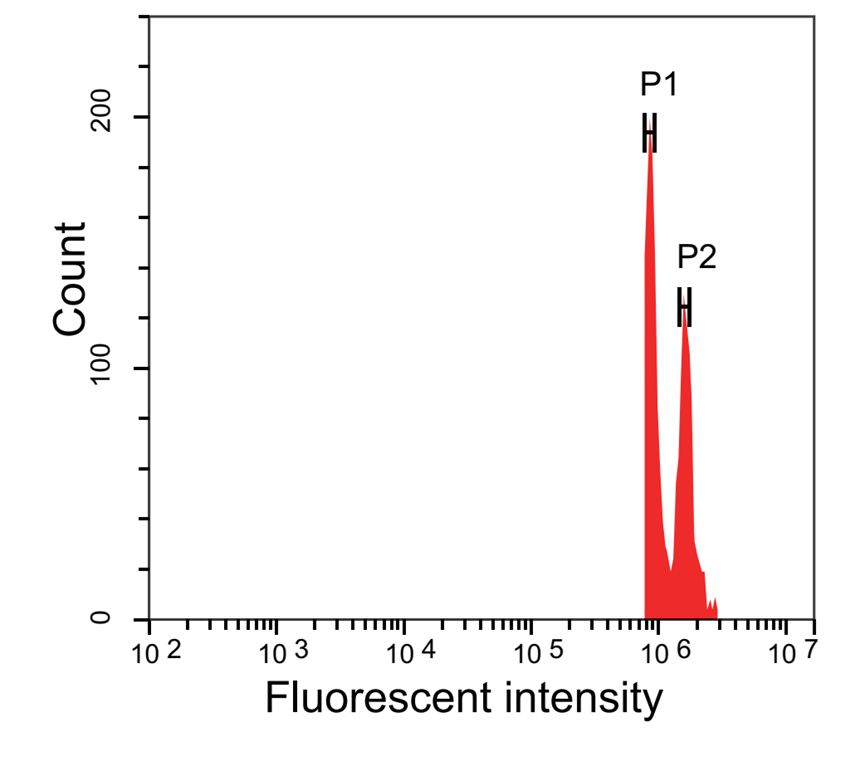


**Figure S1**. Genome size estimation of *Sphingonotus tsinlingensis* using flow cytometry analysis. *Locusta migratoria* is the internal standard. P1 and P2 represent the peaks of *L. migratoria* and *S. tsinlingensis*, respectively. The x-axis indicates fluorescent intensity and the y-axis indicates counts of cells.


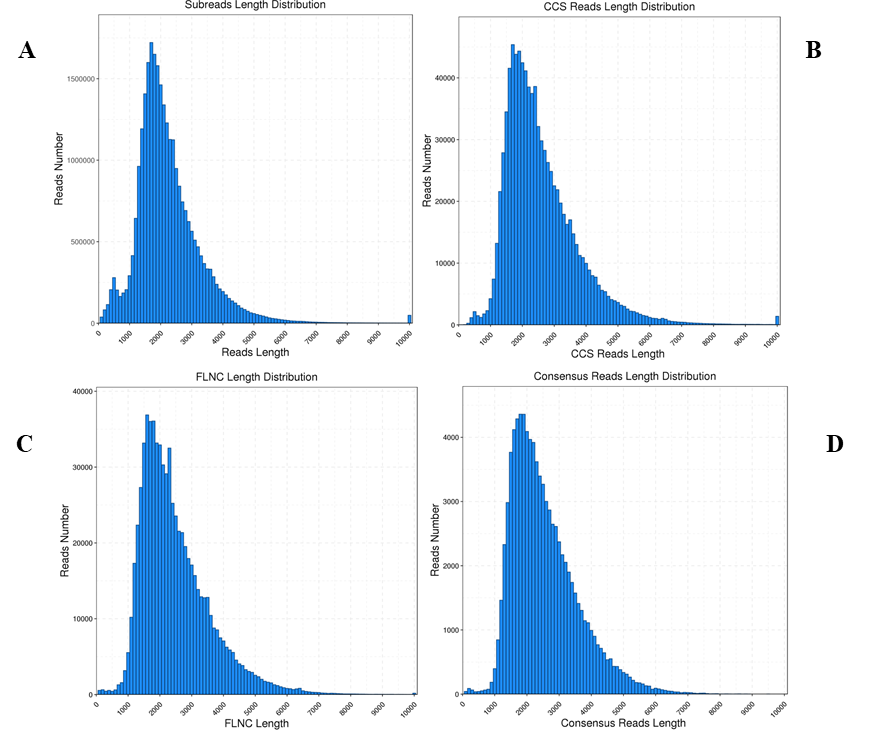


**Figure S2**. Length distribution of clean-reads. A: subreads length distribution; B: circular consensus sequence (CCS) length distribution; C: full-length non-chimeric (FLNC) read length distribution; D: consensus read length distribution.


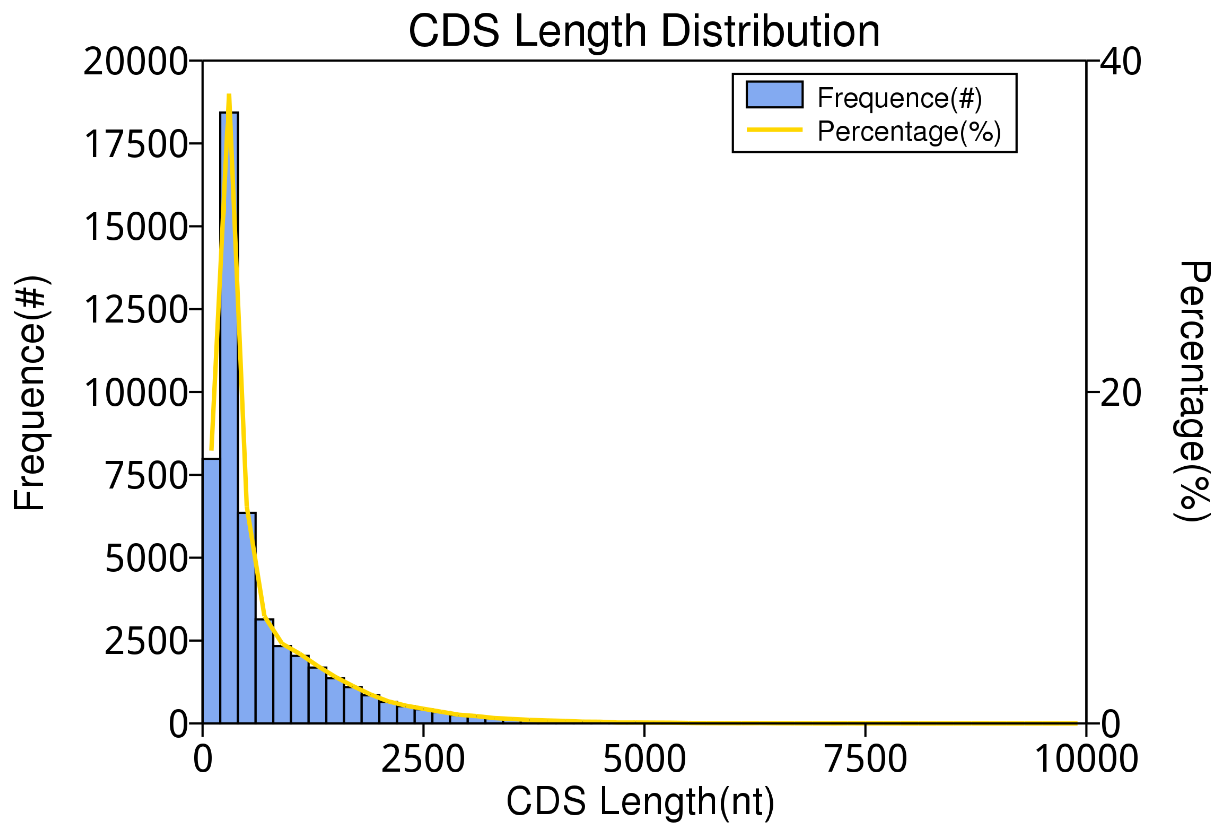


**Figure S3**. Length distribution of all coding sequences.

**(A)**


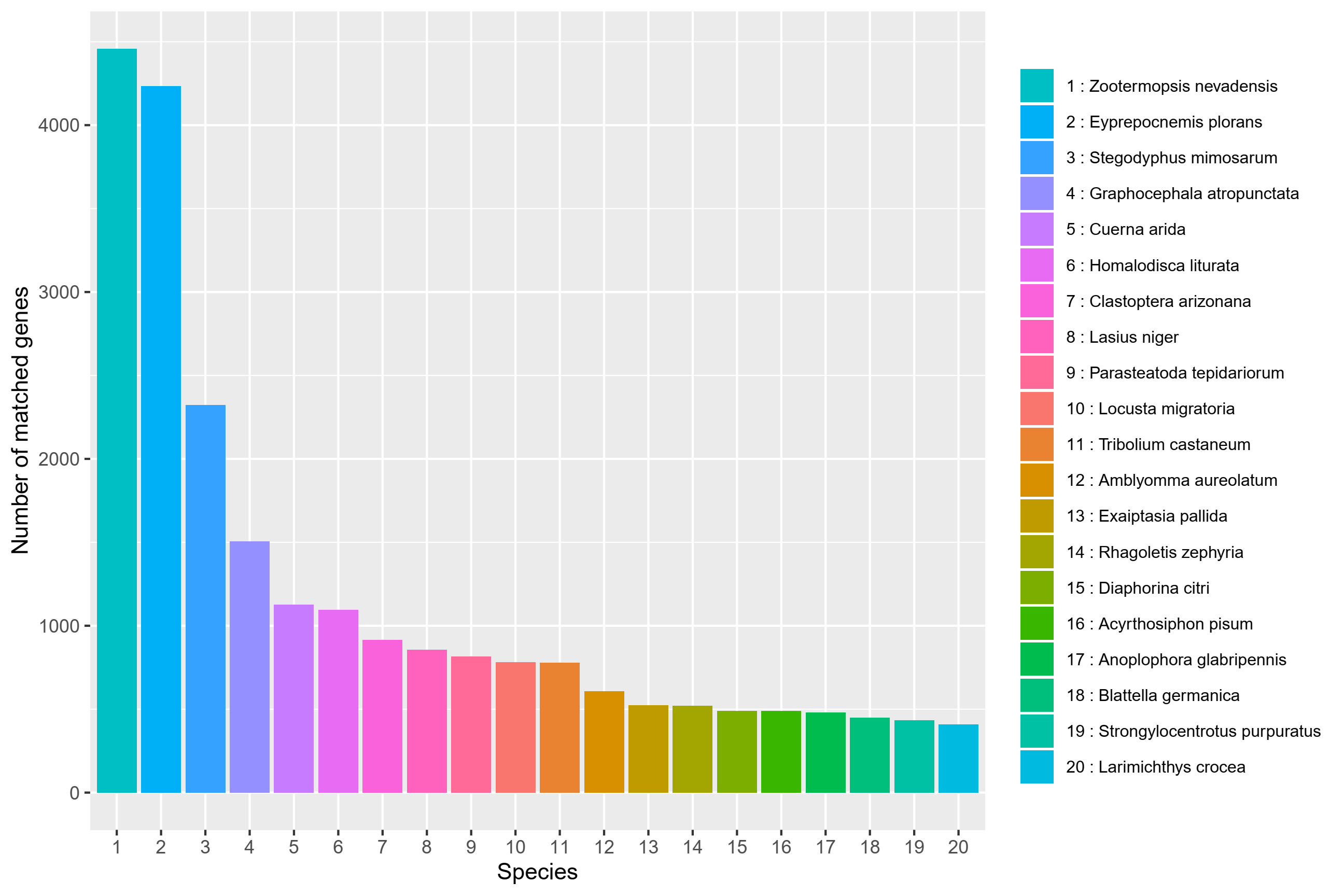


**(B)**


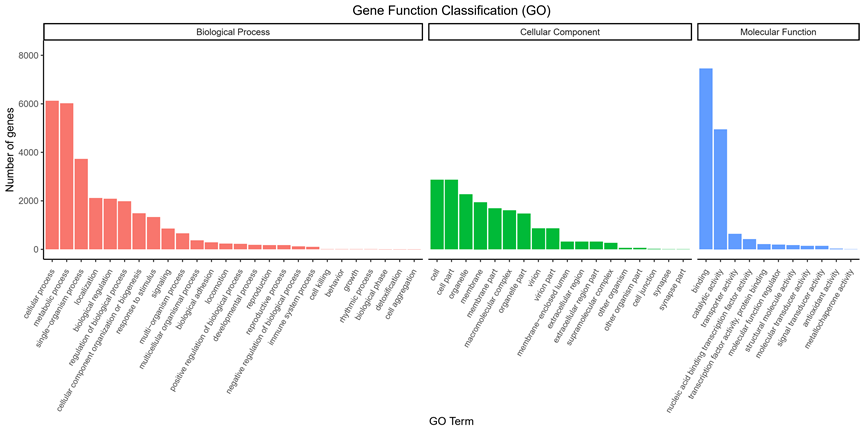


**(C)**


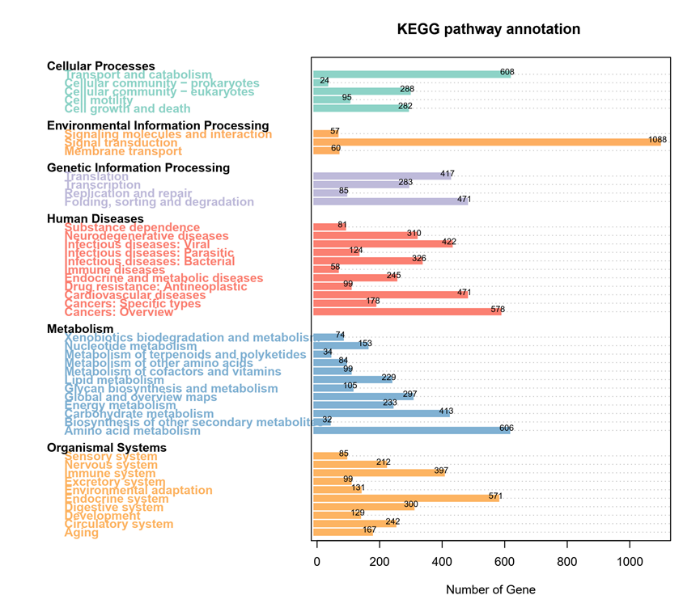


**Figure S4**. Functional annotation using the non-redundant protein sequence (NR) (A), Gene Ontology (GO) (B) and Kyoto Encyclopedia of Genes and Genomes (KEGG) databases (C).


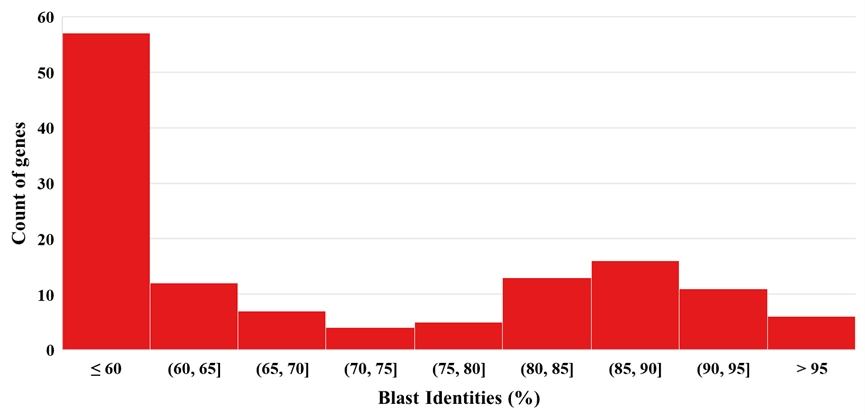


**Figure S5**. Basic local alignment search tool (BLAST) identities of heat shock protein (HSP) and CYP450 genes in the non-redundant protein sequence (NR) database.


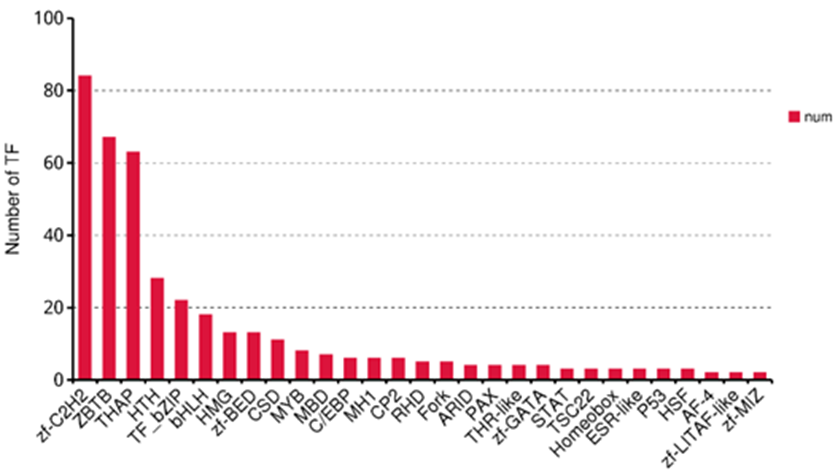


**Figure S6**. Types and number of the top transcription factors (TFs) identified in *Sphingonotus tsinlingensis*.


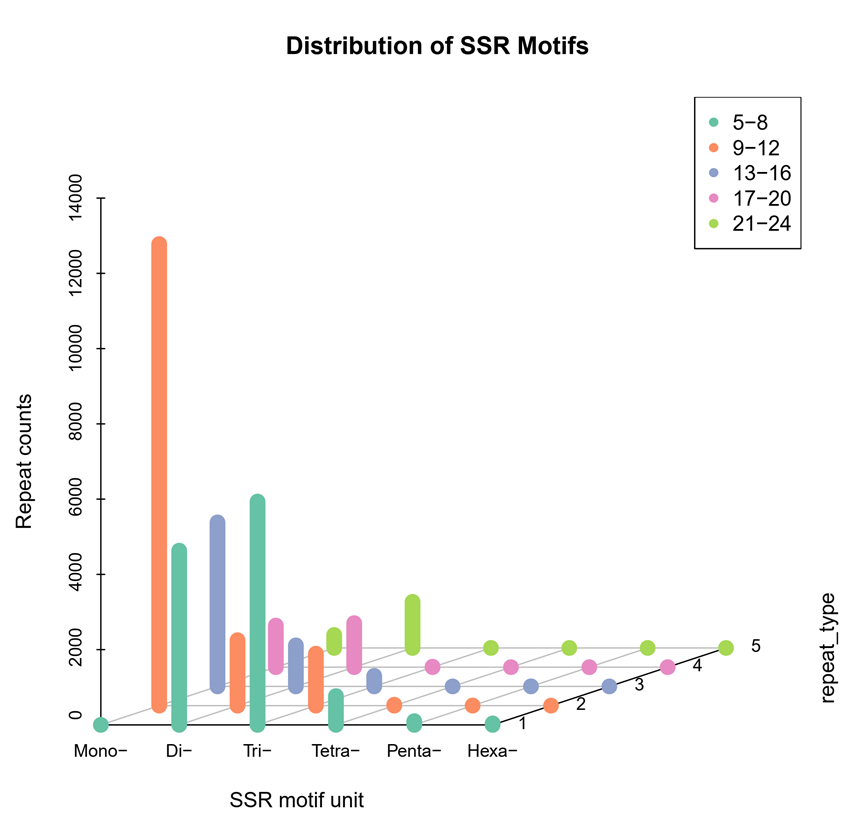


**Figure S7**. Distribution of simple sequence repeat (SSR) motifs in *Sphingonotus tsinlingensis*. The x-coordinate indicates the SSR type, the y-coordinate indicates the repeat type with a specific repetition number, and the Z- coordinate indicates repeat counts.
